# On the detection of carbon fibre storage contamination and its effect on the fibre–matrix interface

**DOI:** 10.1038/s41598-018-34609-y

**Published:** 2018-11-06

**Authors:** Quanxiang Li, Andrea L. Woodhead, Jeffrey S. Church, Minoo Naebe

**Affiliations:** 1 Deakin University, Geelong, Australia, Carbon Nexus, Institute for Frontier Materials, Victoria, 3216 Australia; 2 CSIRO Manufacturing, Waurn Ponds, Geelong, Victoria, 3216 Australia; 3 JPA Scientific, PO Box 2573, Chino Hills, Ca 91709 USA; 4 School of Engineering, Edith Cowan University, 270 Joondalup Drive, Joondalup, Perth, Western Australia 6027 Australia

## Abstract

Contamination caused by inappropriate carbon fibre (CF) storage may have an impact on their end use in reinforced composite materials. Due to the chemical complexity of CFs it is not easy to detect potential contaminants, especially at the early stage during manufacturing and handling. In this paper, X-ray Photoelectron Spectroscopy (XPS), Fourier Transform Infrared (FTIR) spectroscopy and Surface Energy Analysis (IGC-SEA) were used to assess the surfaces of CFs stored in polyolefin zip-lock bags for possible contamination. Only after over 2 months in-bag storage, was XPS capable of detecting a minor increase in nitrogen on the CF surface while FTIR revealed the presence of fatty acid amides and fatty acids, both associated with the storage media. However neither of these techniques were sensitive enough to show significant evolution of the amount of contamination as a function of storage time. In contrast, IGC-SEA distinguished surface energy differences between CFs before and after storage. These differences were found to change as a function of storage time, which were attributed to increases in contamination amounts. Single fibre fragmentation tests indicated that the surface contamination had potential to disrupt the fibre-matrix interface. These findings provide a new method for assessing the surface contamination of CFs with potential application to other materials.

## Introduction

The carbon fibre (CF) surface plays a significant role in determining its end use performance as a reinforcement for composite materials^1–3^. The interface between the fibres and matrix is known to be critical in transferring the stress among fibres through the matrix, distributing the load applied to the composite^4–6^. It is believed that keeping the fibre surface clean is important as the presence of adsorbed contaminants may result in poor interfacial adhesion in the composite system^7,8^.

At the end of the manufacturing process CF tow is wound onto large spools and protected with a polyolefin shrink-wrap. As part of the standard sampling process for CF testing, lengths of CF tow are usually cut from the spools and stored in various containers, including polyolefin zip-lock bags, prior to use. However, in one particular study, evidence was found that inappropriate handling of CF had produced an unwanted interphase between the fibre and the matrix^9^. More importantly, the effect of such an interphase on the performance of a composite is not well characterized, since its precise nature is difficult to predict^7^.

The chemical nature of a CF’s surface is often investigated by Attenuated Total Reflectance (ATR) Fourier Transform Infrared (FTIR) spectroscopy and X-ray Photoelectron Spectroscopy (XPS)^10,11^. These methods provide spatially resolved information on the mm^2^ and 0.1 mm^2^ scales, respectively. While XPS is sensitive to a 10 nm depth, the depth of penetration of a typical ATR experiment can range from 300 nm to several µm. Time-of-flight secondary ion mass spectrometry, a more chemically selective and surface sensitive technique compared to XPS, has also been used to investigate the surface of CFs. It was found that the CF surface was dominated by contamination which served as a history of its manufacturing and handling^12^. Moreover, it is reported in the literature that measuring the wettability of carbon fibre and related materials may indirectly reflect any changes in the material’s surface^13–16^. There are however no significant studies reporting on techniques that can detect potential contamination, especially at the early stage during processing and handling.

A recently developed technique for assessing the surface properties of materials is Inverse Gas Chromatography - Surface Energy Analysis (IGC-SEA)^17–21^. This method has been used to determine the dispersive and specific surface energy distributions of CFs^22,23^. Dispersive energy arises from London interactions while specific energy is largely associated with acid-base interactions. In a study of the surface energy heterogeneity of high, standard, and intermediate modulus CFs^24^ it was found that fibres from different stages of production exhibited different distributions of energy. IGC-SEA was also used to evaluate the adhesion between matrix and fibres by measuring dispersive components and specific interactions of CFs before and after surface modification^2,25^. The acid-base interaction parameter obtained was correlated with the shear strength of the CF interface in composites as determined by single fibre fragmentation tests (SFFT)^26,27^.

In this paper we investigate the capability of a number of techniques, including XPS, FTIR and IGC-SEA, in detecting contamination on the surface of zip-lock bag stored CFs. We extend this investigation through the use of SFFT to assess the effect of surface contamination on the interfacial shear strength (IFSS) of CFs in an epoxy matrix. Initial investigations were carried out on oxidized only CFs as this simplifies the system and thus the interpretation of the results. Finally we apply a similar approach to the analysis of sized CFs, thus providing a practical result with value to the commercial end user.

## Results and Discussion

In the initial phase of this study oxidized but not sized CFs were utilized. While this does not represent the way fibres are typically supplied to end users, their use in this study simplifies the analysis and the approach would be directly transferable to the analysis of the sized CFs.

### Chemical analysis of oxidized carbon fibres

XPS is commonly used in the analysis of CF surfaces^10,28–31^. The XPS survey scan results, expressed as atomic ratio (X/C), obtained from the stored oxidized CF samples are presented in Table S1. No differences in binding energies or elemental composition were detected in the carbon and oxygen peaks of the spool and zip-lock stored CFs. While no differences were observed after 7 days, the surface nitrogen content has however increased after 2 months of zip-lock bag storage. This increase is statistically significant at the 95% confidence level. Trace amounts of sodium, chlorine and silicone were also observed and are likely attributable to minor surface contaminants picked up during fibre manufacturing. After washing with dichloromethane (DCM) the surfaces of both spool and zip-lock bag stored CFs exhibited statistically higher O content relative to C suggesting the removal of a hydrocarbon based material and exposure of the oxidized CF surface.

**Figure 1:**
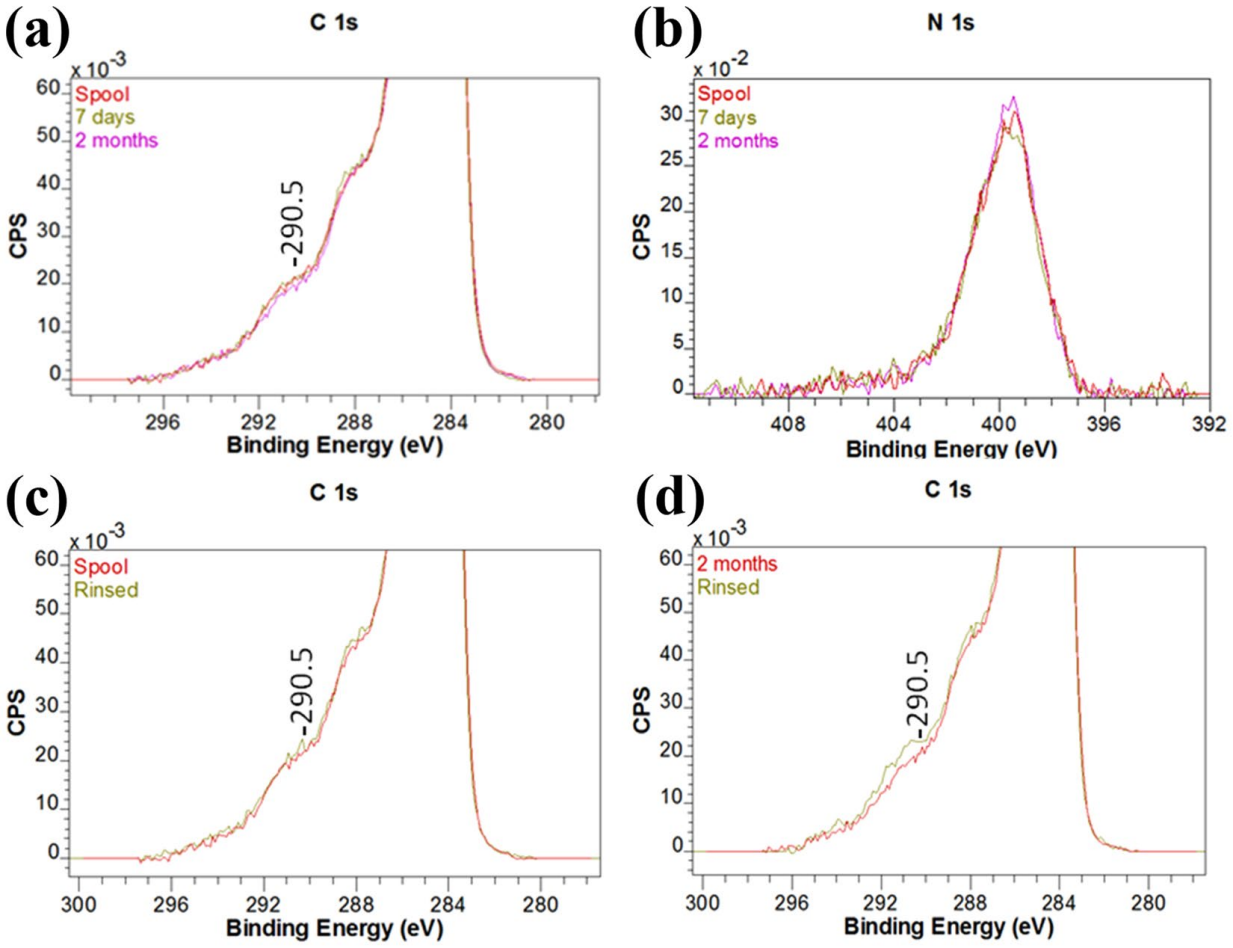
Zoomed in high resolution C 1s (**a**) and N 1s (**b**) XPS spectra obtained from CF from the spool and after storage in a zip-lock bag and high resolution C 1s XPS spectra after rinsing in DCM (**c**,**d**).

Changes in the CF surface due to solvent washing were further investigated by examination of the high resolution C 1s and N 1s scans shown as Fig. 1. XPS spectra obtained from CFs typically have an asymmetric C 1s band with a long tailing component on the high energy side^10^. Determination of the functional groups present on the CF surface relies on the ability to distinguish bands that fall under this tailing.

No differences were observed in the zoomed in C 1s profiles obtained from spool stored CFs and the CFs stored in the zip-lock bag for 7 days (Fig. 1a). Component peaks are observed at 288 and 290.5 eV which are generally assigned to carbonyl (C=O), and carboxylic acid (-COO^−^) groups and π-π* shake up satellites, respectively^32^. After 2 months storage there appears to be a very small decrease in the 290.5 eV component. No statistically significant differences were however observed for the oxygen concentrations detected in the survey scans (Table S1). The N 1s profiles for these samples (Fig. 1b) were also very similar with peak binding energies of 399.78 ± 0.23 eV. A slight intensity increase, consistent with that detected in the survey scans (Table S1), is observed for the 2 month zip-lock bag stored CFs. In all cases a high energy shoulder is observed near 401 eV. In a study of electrolytically oxidized CFs the binding energy component at 399.6 and 399.9 eV were assigned to polyacrylonitrile and polyacrylamide, respectively while components near 401 eV were assigned to poly ether imide and poly allylamine hydrochloride^33^.

The C 1s profiles of the spool and zip-lock bag stored CFs after rinsing with dichloromethane are shown as Fig. 1c,d, respectively. Rinsing had no significant effect on the spool stored fibres but in contrast more carboxylate groups appear to be exposed on the surface of the zip-lock bag stored fibres. This finding is supported by the small but significant increase in oxygen concentration detected in the survey scan (Table S1). Similar N 1s profiles were obtained for the CFs both before and after DCM rinsing.

**Figure 2:**
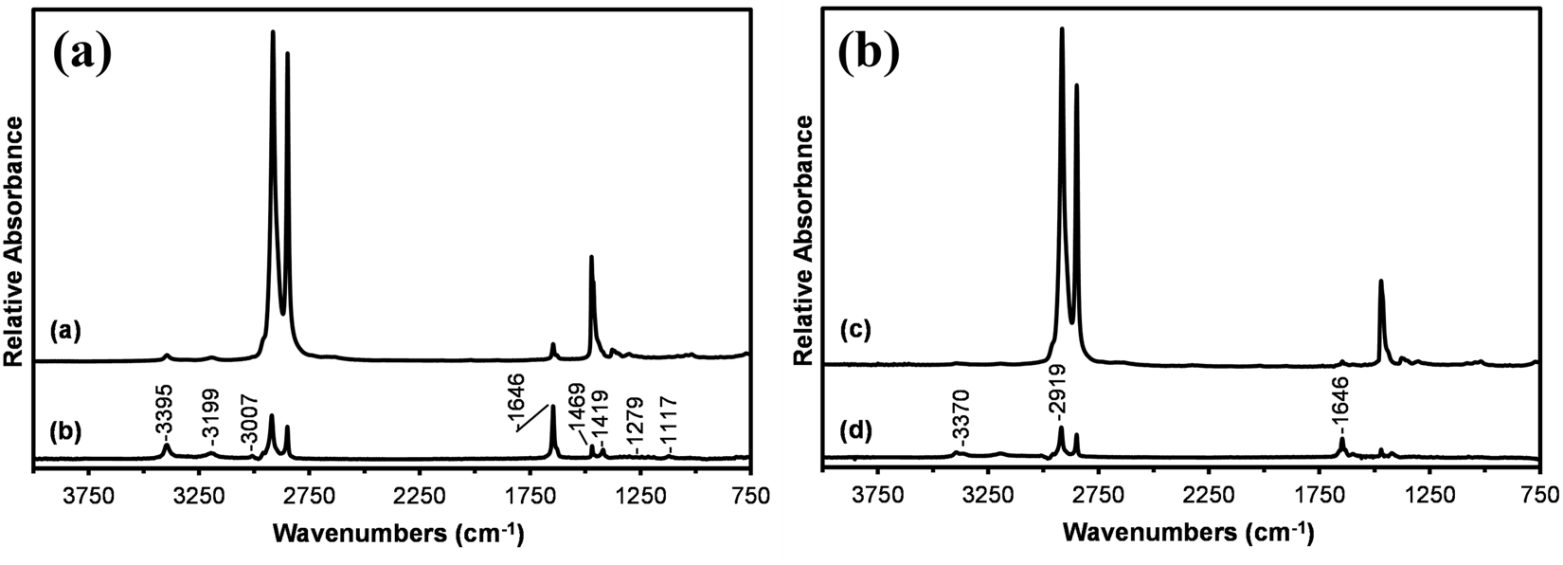
ATR infrared spectra obtained from the storage media: (**a**) zip-lock bag used to store the CFs (**b**) residue left by the bag and (**c**) shrink-wrap used to protect spools of CF during storage and (**d**) residue left by the shrink-wrap.

FTIR ATR spectroscopy has the potential to provide more detailed chemical information compared to XPS as its functional group peaks are often better resolved. The depth at which data is obtained is however 30 to 50 times greater (see Supporting information, Appendix A for details) and thus this approach for the surface analysis of the CFs was not pursued. Based on the XPS results however it was decided to investigate the surfaces of the two storage media. The ATR spectra obtained from the surfaces of the zip-lock bag and shrink-wrap film are shown in Fig. 2a,c, respectively.

These spectra are consistent with that of polyethylene however the observation of weak bands above 3000 cm^−1^ and near 1650 cm^−1^ is indicative of the presence of an additive or surface active material. Based on relative intensities there is less of this material present on the surface of the shrink wrap film. Transmission spectra obtained from the two storage media (Figure S1) confirmed that these weak spectral features were not associated with co-polymers.

Often a surface active material will leave a residue on the ATR crystal making it possible to obtain a spectrum of this material without interference from the substrate^34^. The spectra obtained from these residues, shown as Fig. 2b,d, are in excellent agreement with that of the fatty acid amide erucamide (erucamide, *cis*-13-docosenamide) with major peaks at 3395, 3199, 3007, 1646, 1469, 1419, 1279 and 1117 cm^−1 ^^35^. Erucamide is typically added into the formulation of films as a slip agent and migrates to the film surface over time. The bands observed near 3395 and 3199 cm^−1^ in Fig. 2b,c are assigned respectively to the asymmetric and symmetric NH_2_ stretching modes of the primary amide erucamide^36^. Close examination of the residue left from the shrink wrap surface, Fig. 2d, reveals an additional feature at 3370 cm^−1^ that can be assigned to an N-H stretching mode. Further, the 1646 cm^−1^ amide band is significantly weaker relative to the 2919 cm^−1^ CH_2_ vibration compared to that observed for the zip-lock bag, Fig. 2b. Both of these differences point to the presence of a secondary fatty acid amide such as erucyl erucamide in the shrink wrap film. This higher molecular weight compound has a lower migration rate.

**Figure 3:**
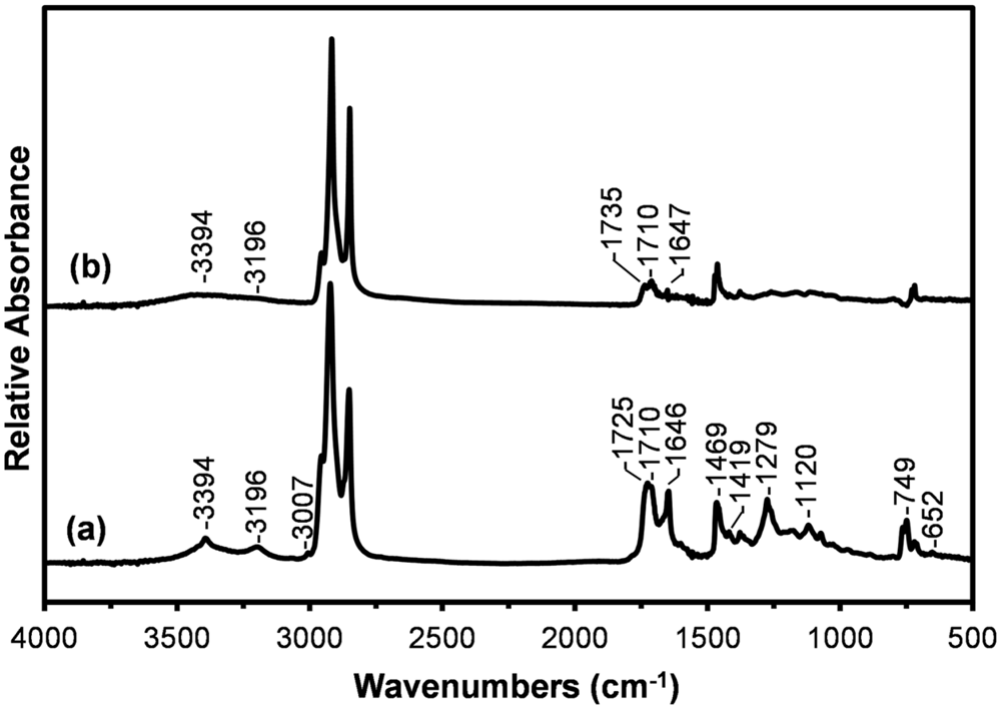
Infrared transmission spectra of thin films cast from the DCM rinse residues obtained from (**a**) CFs after 2 month storage in a zip-locked bag and (**b**) CFs from the spool.

One approach for increasing the detection limits of infrared spectroscopy for the detection of CF surface contamination is to isolate and concentrate any surface active material present on the fibre surface by rinsing with an appropriate solvent. By using a large mass (~1 g) of fibre it was found that residues were visibly apparent after solvent evaporation. In contrast, the evaporation of the same amount of solvent (a blank) showed no residue. The infrared spectra obtained from the films cast from the residues taken up in a few drops of solvent are shown in Fig. 3.

The spectrum obtained from the residue rinsed from CFs stored in a zip-lock bag for 2 months (Fig. 3a) appears to be a mixture of two materials, both likely dominated by long alkyl (-CH_2_-) chains. There is clear evidence of the presence of a fatty acid amide through the NH_2_ and amide features which are in good agreement to that detected on the surface of the zip-lock storage bag (Fig. 2b). In addition to the fatty acid amide features there is a strong C=O stretching band with components at 1725 and 1710 cm^−1^. The 1725 cm^−1^ feature is possibly associated with a conjugated ester while that at 1710 cm^−1^ is consistent with that expected for an acid dimer^36^.

The spectrum obtained from the film cast from the residue rinsed from the spool fibres (Fig. 3b) can be identified as that of a mixture of saturated fatty acid esters and the hydrolyzed acids appearing as acid dimers (1735 and 1710 cm^−1^, respectively)^36^. Weak features are also observed at 3394, 3196 and 1647 cm^−1^ which can be associated with the presence of a fatty acid amide. Clearly the surface of the shrink-wrap protected CFs is contaminated with an organic material but not to the extent of the zip-lock bag stored CFs.

The fatty acid amide molecule is amphiphilic, being comprised of a mono-unsaturated aliphatic chain terminated with a polar amide group. The mono-unsaturation creates a bend in the aliphatic chain which inhibits molecular packing. The presence of fatty acid amides and fatty acid esters/acids on the CF surface has the potential to disrupt interactions at the fibre-matrix interface in composites. It is also possible that they could migrate into the epoxy changing its physical properties.

From the above results it is evident that XPS and FTIR cannot detect the presence of contamination unless there are significant extraneous molecules on the fibre surface. These techniques are not sensitive enough to show significant evolution of the amount of contamination as a function of time. When carrying out IGC–SEA at finite concentration conditions, the probe molecule will preferentially attach to the most reactive sites, the measured surface energy as a function of fractional surface coverage reveals information about the range of the surface energies of the different sites on the sample, thus making the technique very sensitive to small changes. The technique thus provides graphs of surface energy, dispersive, specific or acid–base, and total, as a function of surface coverage. Energetically homogeneous surfaces are indicated by a flat line. What is more commonly observed is an exponentially decaying surface energy function which is indicative of surface heterogeneity.

**Figure 4:**
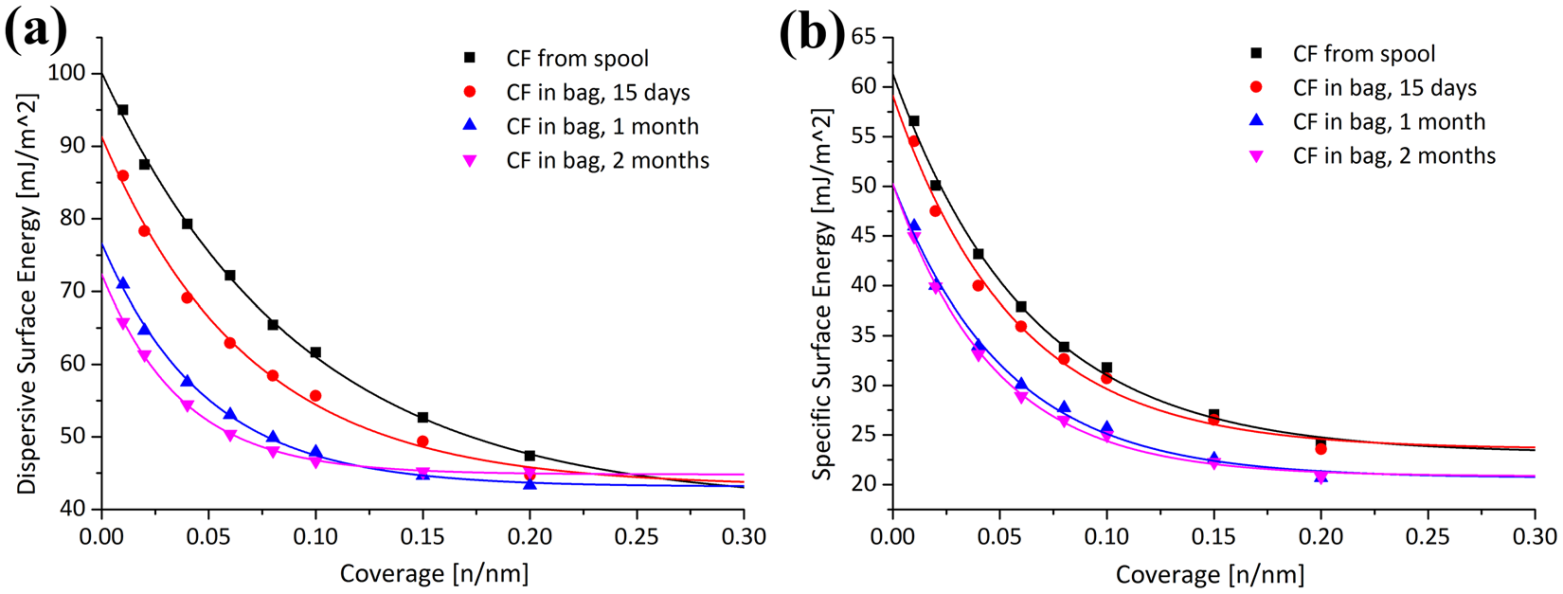
Time sequences depicting the change in dispersive surface energy (**a**) and specific (acid–base) surface energy (**b**) for the oxidized CFs pre-conditioned at 30 °C.

IGC-SEA of a sample is preceded by a conditioning pre-treatment to remove any physisorbed molecules such as water. The presence of these molecules, which would occupy the highest energy sites, could lead to artificially lower surface energy values. Pre-treatment generally involves heating the sample under helium flow to just above 100 °C. As CFs are stored at room temperature in an environment which contains water vapour and would be used without pre-treatment, the initial IGC-SEA measurements were carried out using the mild pre-conditioning temperature of 30 °C. Based on Schultz method^37^, the surface energy of a solid can be linked to the retention volume of a probe. As an example, the calibration curves obtained by using the different probes at 0.01 n/nm coverage are shown in Figure S3. Surface coverage is defined as the number of moles adsorbed (n) divided by the number of moles required to create a theoretical monolayer surface coverage (nm). Therefore, the n/nm values represent a fractional surface coverage ranging from 0 (i.e. infinite dilution) to 1.0 (i.e. monolayer coverage). The dispersive, ${\gamma }_{S}^{D},$ and specific (acid-base), ${\gamma }_{S}^{{AB}},$ surface energy profiles of the CFs obtained as a function of storage time are shown in Fig. 4.

The fibres cut from the spool are energetically more active and more heterogeneous compared to the zip-lock bag stored fibres. These profile differences were found to be a function of zip-lock bag storage time with the largest decreases occurring in the first month. Only a relatively small change was observed from this point until the 6 months end of the experiment (Figure S4). The parameters obtained from the exponential fitting of the profiles obtained from CFs stored up to 2 months are presented in Table 1. For completeness the fitting results for the total surface energies, ${\gamma }_{S}^{T}$, are also given.

**Table 1:** Parameters obtained from fitting an exponential decay function; y = y_0_ + Ae^x/t^, to the surface energy data of the stored CFs conditioned at 30 °C.

Energy Type	CF Storage	Energy (mJ m^−2^)			Decay Constant (t)
		Average y(1)	Maximum y(0)	Range (A)	
Dispersive(${{\boldsymbol{\gamma }}}_{{\boldsymbol{S}}}^{{\boldsymbol{D}}}$)	Spool	40.7	100.2	59.5	0.093
	Bag, 15 days	43.2	91.3	48.1	0.069
	Bag, 1 month	45.0	77.0	32.0	0.046
	Bag, 2 months	44.0	72.1	28.2	0.042
Specific (${{\boldsymbol{\gamma }}}_{{\boldsymbol{S}}}^{{\boldsymbol{AB}}}$)	Spool	23.1	61.4	38.2	0.063
	Bag, 15 days	23.6	59.2	35.6	0.057
	Bag, 1 month	21.2	49.4	28.2	0.046
	Bag, 2 months	18.1	43.7	25.6	0.047
Total (${{\boldsymbol{\gamma }}}_{{\boldsymbol{S}}}^{{\boldsymbol{T}}}$)	Spool	64.7	161.2	96.5	0.079
	Bag, 15 days	67.0	150.4	83.5	0.063
	Bag, 1 month	66.1	126.3	60.2	0.046
	Bag, 2 months	62.1	115.8	53.7	0.044

It was found that the average dispersive surface energies (y(1)) are similar for all samples, 43.2 ± 1.8 mJ m^−2^, and during this time the maxima (y(0)) for the dispersive decrease by ~28%. The decay constants also decrease indicating a more homogeneous surface is being attained with increased storage time. These changes provide insight into the mechanism driving the changes in surface heterogeneity observed. They indicate that some foreign materials, i.e. contaminants, are occupying the highest dispersive component of the surface energy. With time going on, increasing highly energetic energy sites on the carbon fibre surface are being covered by contaminants from the bag, and only the sites with relatively low surface energy are maintained. As a result, the energy value of the most energetic energy sites (infinite dilution - 0.3 n/nm coverage) decrease while the low energy sites (0.3 - 1 n/nm coverage) are unchanging.

**Figure 5:**
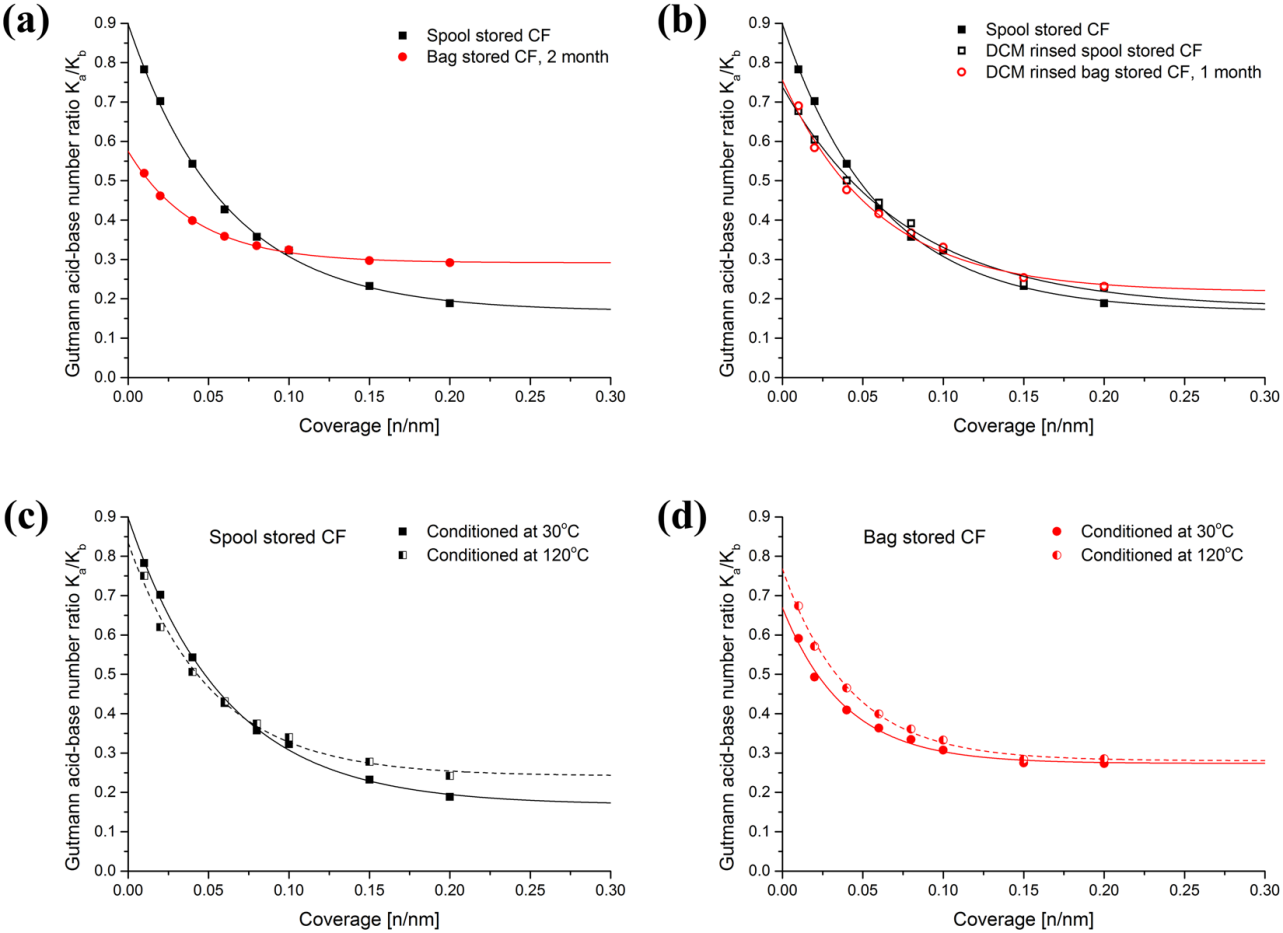
Plots of the Gutmann acid-base number ratio as a function of surface coverage for (**a**) spool and 2 month zip-lock bag stored oxidized CFs, (**b)** DCM rinsed spool and 1 month zip-lock bag stored oxidized carbon fibres (the profile for the spool stored fibres prior to rinsing is also shown for comparison). All fibres were pre-conditioned at 30 °C. The lower plots compare the effect of conditioning temperature for spool stored oxidized carbon fibres (**c**) and 1 month zip-lock bag stored fibres (**d**).

The average specific surface energy appears to reduce as a function of storage time by 22%. The average surface becoming less polar can be confirmed from the ${\gamma }_{S}^{{AB}}/{\gamma }_{S}^{T}$ value dropping from 0.36 to 0.29. The maxima (y(0)) and decay constants for the specific energies follow a similar trend as the dispersive surface energy. As the Gutmann acid-base number ratio values for the spool stored fibres are less than 1 (Fig. 5a), the surface is generally more basic in nature. This is consistent with previous findings^24,38^. After 2 months zip-lock bag storage the surface was found to become less basic on average.

In order to avoid any water vapour influence and further explore the surface differences between the stored CFs, IGC-SEA was carried out at higher conditioning temperatures. These results are presented in Table S2. When the oxidized fibres were conditioned at 120 °C it was found that the surface energy for both CFs became more similar to each other. But the maximum energy value for the spool stored CFs is still significantly higher than that of zip-lock bag stored CFs, and is more sensitive to temperature than that of the zip-lock bag stored CFs. The change in the Gutmann acid-base number ratio also showed a different trend (Fig. 5c,d). The average value of acid-base ratio of spool stored fibre increased to 0.25 while there was no significant change for bag stored CF. In contrast, the maximum of the zip-lock bag stored fibres increased in value similar to that of the spool stored fibres after 120 °C conditioning. The overall change in surface energy and acid-base status could possibly be attributed to a combination of the loss of physisorbed polar molecules and a redistribution of foreign material on the fibre surface. The behaviour upon conditioning of the spool and zip-lock bag stored CFs suggests that their surfaces are significantly different.

To further understand the surface differences between the CFs, they were rinsed with DCM and dried at room temperature. The IGC-SEA results revealed that after rinsing, both spool and zip-lock bag stored fibres had very similar ${\gamma }_{S}^{D}$ and ${\gamma }_{S}^{{AB}}$ profiles (see Table S2). Compared to the original spool stored fibres the rinsed fibre profiles have higher average total energy (~21%) and are more homogeneous. While the maximum values of both specific and dispersive energies increased, the greater increase was in the dispersive energy, to 112 mJ m^−2^, which is within the range, 120 to 90 mJ m^−2^, reported for a graphitic plane^38^. This increase is consistent with the exposure of more non-polar surface sites. These changes also give rise to a small decrease in the average surface polarity, ${\gamma }_{S}^{{AB}}/{\gamma }_{S}^{T}$. Clearly the surface of the shrink-wrap protected CFs is contaminated with an organic material but not to the extent of the zip-lock bag stored CFs. This is consistent with the FTIR results. But these results are in contrast to the XPS findings where rinsing had no significant effect on the spool stored fibres and can possibly be explained in terms of the differences in surface sensitivity of the two techniques. Further discussion on the sensitivity of XPS is presented in the Supporting information as Appendix B.

The Gutmann acid-base number ratio plots for the spool stored and 1 month zip-lock bag stored fibres after rinsing with DCM (Fig. 5b) are also found to be very similar. Compared to the profile obtained for the original spool stored fibres, also shown in Fig. 5c,d, the corresponding rinsed fibres (Fig. 5b) have a lower maximum but similar average value and decay constants. The DCM rinsing process has therefore exposed more high energy basic sites. From the IGC-SEA results, the DCM rinsing process appears to have removed material from the surfaces of the zip-lock bag stored fibres.

### Physical assessment of oxidized carbon fibres

To assess the influence of the surface contamination on interfacial bonding, the IFSS of CFs in an epoxy matrix is determined by means of single fibre-composite fragmentation. The morphology associated with the fibre breaks can offer valuable clues regarding the interface strength^39^. A stronger bond between fibre and matrix usually results in a shorter critical fragment length. Furthermore, aspects such as the shape of fibre breaks and the debonding characteristics between fibre and matrix can provide a wide range of information about the adhesion for a test specimen^40^. The birefringence pattern around the fibre crack region is caused by the interfacial shear and frictional stresses and strains at the interface^41^. The interfacial shear strength results obtained for spool and zip-lock bag stored CFs both before and after DCM rinsing are presented in Table 2.

**Table 2:** Single fibre-composite fragmentation results for stored oxidized CFs.

CF Storage and Pre-treatment	Tensile Strength (cN dtex^−1^)		Weibull Modulus	Fragment Length (µm)		${{\boldsymbol{\tau }}}_{{\boldsymbol{IFSS}}}$ (cN dtex^−1^)
	Mean	SD		Mean	SD	
Spool	21.75	3.58	7.21	463.18	109.59	0.206
Bag, 6 months	20.21	3.12	7.31	455.15	122.18	0.194
Spool, DCM rinsed	21.24	3.33	7.43	473.45	100.82	0.193
Bag, 6 months, DCM rinsed	22.07	3.56	7.50	449.13	101.74	0.212

**Figure 6:**
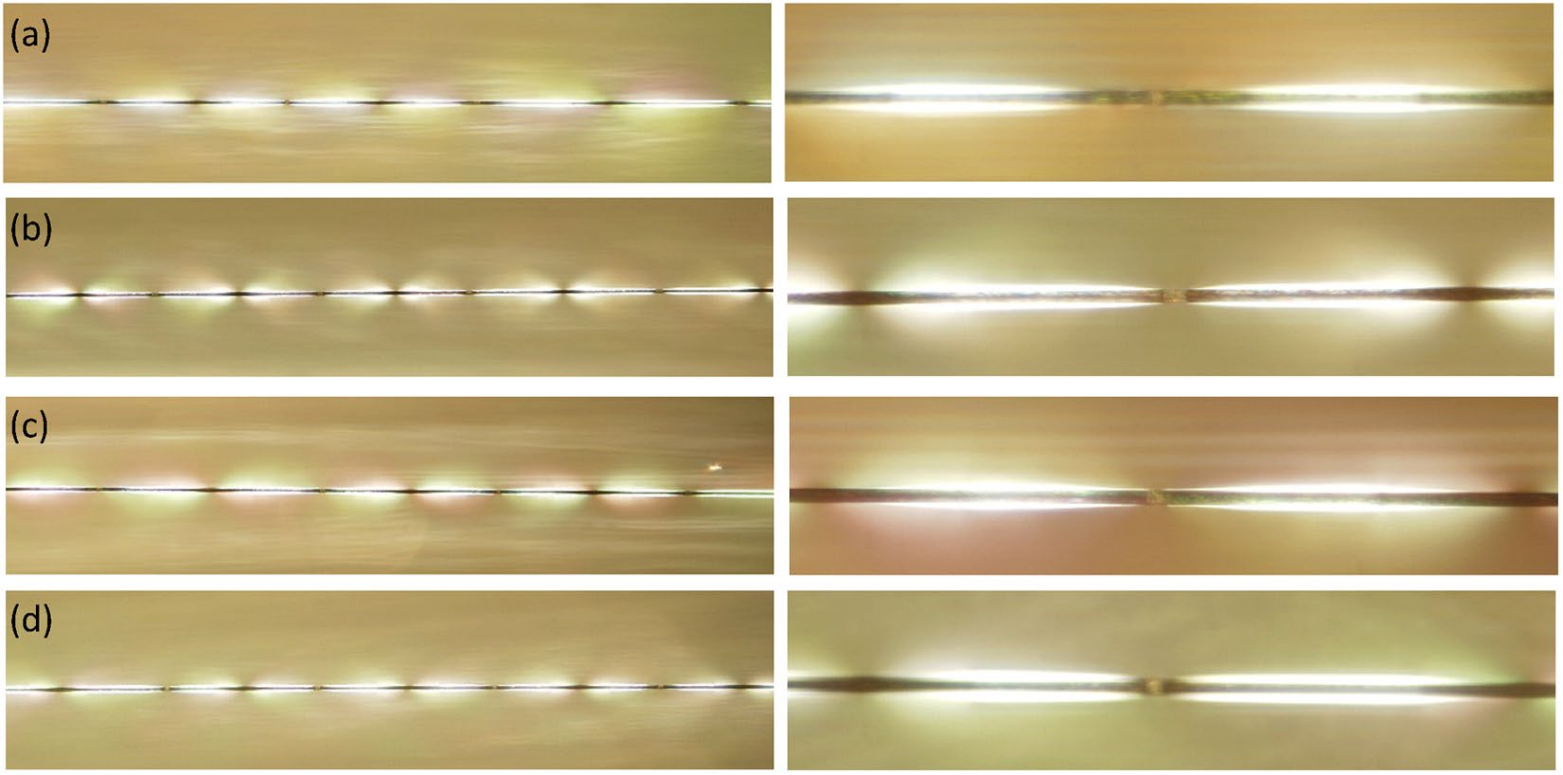
Overall view (left) and fibre break point (right) of the birefringent stress patterns observed for single fibre composites (**a**) CF from spool (**b**) CF in bag 6 months (**c**) CF from spool, DCM rinsed and (**d**) CF in bag 6 months, DCM rinsed.

There is no significant difference in fragment length and resulting interfacial shear strength, ${\tau }_{{IFSS}}$, based on a 2 tailed t-test (n > 50). But the smaller standard deviation (SD) of the fragment length might indicate that the CF’s surface properties have become more uniform after DCM rinsing. Typical birefringent interfacial stress patterns for the four single fibre composites are shown in Fig. 6. All four CF composites exhibited similar densities of birefringent patterns. However, by focusing on the fibre break points, some but not all break gaps between two fibre fragments within the composite prepared from the bag stored fibre appear larger (Fig. 6b, right). The standard deviation of the fragment length was also larger for this CF. Both of these could result from loosely bonded or weak interfaces^40,42^.

### Chemical analysis and physical assessment of sized carbon fibres

As oxidized only CFs are not provided commercially or utilized in industry, the study was extended to sized CFs. While the results have the potential to be more complicated, they would be more meaningful in the applied sense.

**Figure 7:**
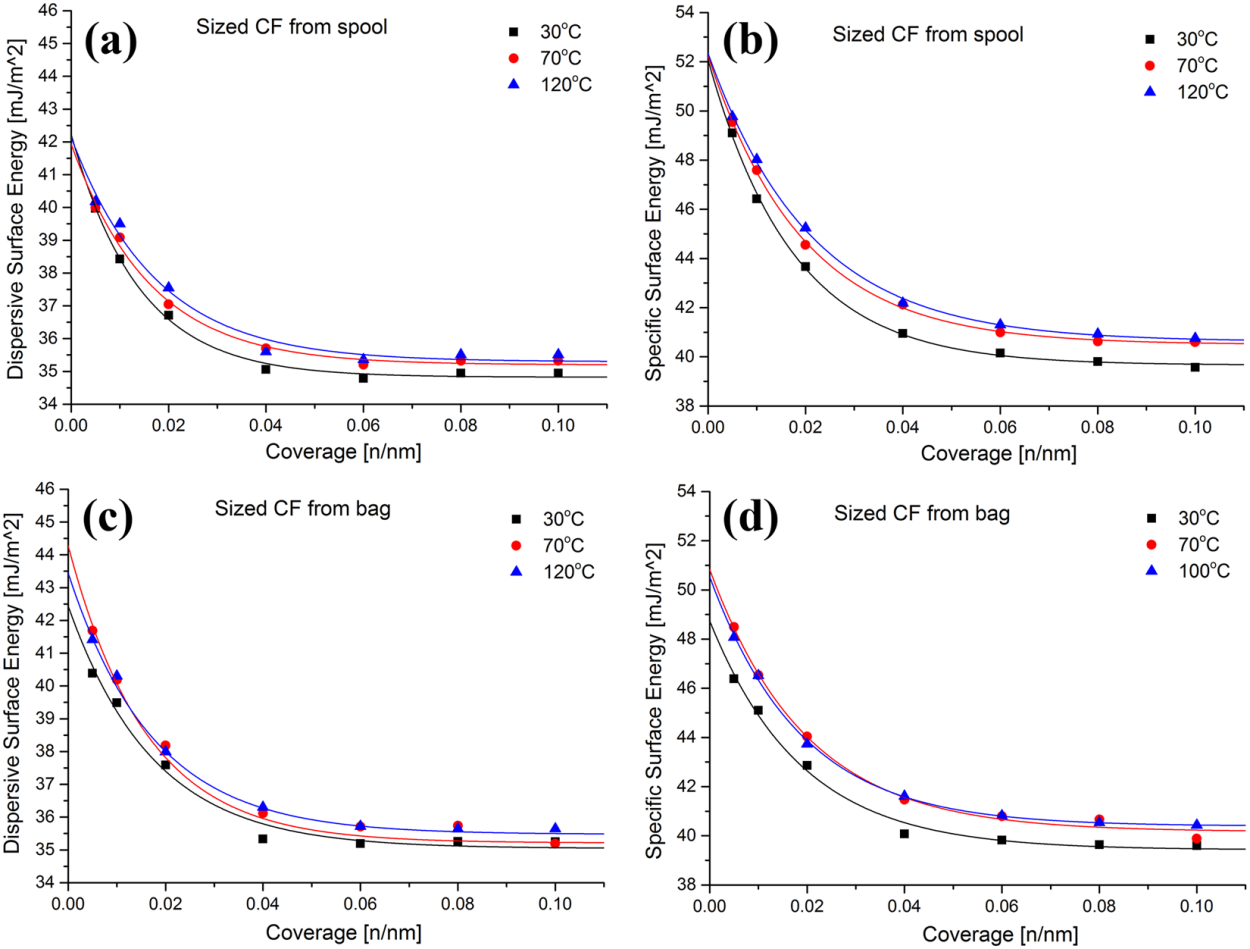
The dispersive energy (**a**,**c**) and specific (acid–base) energy (**b**,**d**), for spool (top) and zip-lock bag (bottom) stored sized CFs as a function of surface coverage.

The dispersive and specific energies as a function of surface coverage for the sized CFs stored on the spool and for 2 months in a zip-lock bag are shown as Fig. 7. The effects of three different pre-treatment temperatures, 30, 70 and 120 °C, are shown.

Compared to the oxidized only fibres, the two different storage methods had little effect on the dispersive surface energies profiles obtained from the sized CFs. The pre-treatments also appear to have little effect. The specific surface energy results are also very similar to each other. The 70 and 120 °C pre-treated fibres however exhibited slightly higher energy, consistent with the removal of small molecules from the fibre surface. These results suggest that the presence of any transferred surface contamination is not significantly affecting the surface energy of the sized fibres.

In an attempt to detect the presence of fatty acid amides or other contaminates on the spool and zip-lock bag stored sized CFs, ~1 g of each were rinsed in DCM solvent. In both cases, significant amounts of residues were isolated after solvent evaporation. The infrared spectra obtained from films cast from these residues (Figure S2) were found to be very similar and identified as those of a bisphenol A based epoxy resin with an ester component, and likely to be the sizing. No evidence of a fatty acid amide could be detected suggesting that it is present is a minor component compared to that of the epoxy size. Based on the IGC-SEA and infrared results it is likely that any contamination, including fatty acid amides esters and acids, present on the surface of the sized CFs has been absorbed into the epoxy sizing.

The interfacial shear strength values obtained for stored sized CFs are presented in Table S3. The results indicate that there is no significant difference in fragment length and resulting interfacial shear strength. More interestingly, the fragment length standard deviation values have narrowed compared with those of the correspondingly stored oxidized CFs. These results are in agreement with those obtained by IGC-SEA and suggest that any contamination, including fatty acid amides, present on the surface of the stored sized CFs has been absorbed and dispersed into the epoxy sizing where it has little if any effect on the fibre-matrix interfaces.

### Probable solutions

Using other clean packaging material to replace zip-lock bag and shrink-wrap film would be the most effective way to avoid contamination on CFs. Based on our experience, glass container which were rinsed with several pure solvents of different polarities and clean paper bag that were not surface treated are ideal to store fibre samples in the lab, especially for carbon fibre surface treatment study and interface property measurement aim. However, shrink-wrap films are still the most convenient and cost-effective packaging material for the current carbon fibre production industry. Because the shrink-wrap films are relatively clean and only makes contact with the outermost CFs before being removed immediately prior to use, the effect of contamination on the whole fibre spool (~3 kg), especially on sized CFs is limited. However, we feel that by the presence of a single layer of clean paper to separate the carbon fibre and shrink-wrap film could be useful to either prevent or further limit the transfer of organic contaminants.

## Conclusions

This work has established the presence of organic contamination on the surface of CFs that were stored in zip-lock bags. The level of contamination was shown to build as a function of storage time until a saturation level was reached. Compared with XPS and FTIR analysis which could only detect this contamination after a relatively long period of storage time, IGC-SEA was able to detect this kind of contamination at a much earlier stage. Through the use of IGC-SEA, variations in surface energy heterogeneity as a function of storage time were easily detected. Moreover, it was shown that the Gutmann acid-base number ratio measured by IGC-SEA has the potential to provide more guidance for understanding the interaction between CFs and the polymer matrix in future composite work.

The results of single fibre fragmentation tests indicated that the surface contamination detected has potential to disrupt interactions at the fibre-matrix interface in composites. Although the level and type of contamination detected in this study appears to have no significant effect on the performance of the sized CFs in a composite, the high sensitivity of IGC-SEA, arising from its use of molecular level probes, enhances the likelihood of the early stage detection of contamination and thus the prevention of this foreign material making it into final products.

The IGC-SEA technique also provides highly useful information about the sample surface which can be used to solve often difficult materials problems such as delamination and adhesion. One may expect further application of IGC-SEA in the examination of materials for surface contamination.

## Materials and Methods

### Materials

Oxidized as well as oxidized and sized intermediate modulus (242 GPa) polyacrylonitrile based 50 K automotive grade CFs (Panex 35) were supplied by Zoltek Hungary. The spools of fibre were protected by a polyolefin shrink-wrap typical of commercial CFs and handled using 100% nitrile powder free gloves. All solvents and IGC-SEA probes were of chromatographic grade (Sigma-Aldrich) and used as received.

### Treatments

CF tows taken from the spools were stored in polyolefin zip-lock bags. During this time small samples were removed for analysis. ~1000 mg of tow from the spools and the zip-lock storage bags were rinsed in 30 mL of HPLC grade dichloromethane (DCM, ≥99.9%, Sigma-Aldrich Australia), dried at room temperature and stored in solvent cleaned glass beakers covered with watch glasses. The DCM rinse liquors were allowed to evaporate at room temperature to isolate any residues.

### Characterization

X-ray photoelectron spectroscopy analysis was performed using an AXIS Ultra DLD spectrometer (Kratos Analytical Inc., Manchester, UK). Data processing was performed using CasaXPS processing software version 2.3.15 (Casa Software Ltd., Teignmouth, UK).

Infrared spectra were obtained at a resolution of 4 cm^−1^ using a Perkin Elmer (Beaconsfield, UK) System 2000 FTIR spectrometer. Spectral data manipulation was carried out using Grams AI v 9.1 (Thermo Fischer Scientific, USA).

Surface energy and specific surface area determination was carried out using an IGC-Surface Energy Analyser (Surface Measurement Systems, Alperton, Middlesex, UK).

The preparation of test specimens for single fibre-composite fragmentation testing is described in the supplementary section. Each specimen was strained up to 6% to ensure crack saturation^43^ at a crosshead speed of 0.05 mm min^−1^ using an Instron 5967 tensile tester (Instron, USA).

Fibre tensile strength as utilized in the SFFT was determined for individual fibres, >70 measurements for each CF sample, using a Favimat + Robot 2 single fibre tester (Textechno H. Stein, Germany). To characterize variations in CF tensile strengths before and after rinsing with DCM, the single fibre tensile results were further analysed using a two-parameter Weibull distribution^44^.

Further specific details covering data collection and analysis for the test methods used are presented in the supplementary section.

## Electronic supplementary material

Supplementary Information
