# Supplementary material for: On the detection of carbon fibre storage contamination and its effect on the fibre–matrix interface
Source: Sci Rep. 2018 Nov 6;8:16446. doi: 10.1038/s41598-018-34609-y (PMC6219532; doi:10.1038/s41598-018-34609-y)
Supplement: Supplementary file 1 — Supplementary Information [file 41598_2018_34609_MOESM1_ESM.docx]

**Supporting Information**

**On the detection of carbon fibre storage contamination and its effect on the fibre–matrix interface**

Quanxiang Li^a*^, Andrea L. Woodhead^b^, Jeffrey S. Church^b,c^, Minoo Naebe^a,d*^

*^a^**Deakin University, Geelong, Australia, Carbon Nexus,* *Institute for Frontier Materials., Victoria 3216, Australia*

*^b^**CSIRO Manufacturing, Waurn Ponds, Geelong, Victoria 3216, Australia*

*^c^JPA Scientific, PO Box 2573, Chino Hills, Ca 91709, USA*

^d^ *School of Engineering, Edith Cowan University, 270 Joondalup Drive, Joondalup, Perth, Western Australia 6027, Australia*

**Experimental Details**

XPS

XPS analysis was performed using a monochromated Al K_α_ source at a power of 144 W (12 kV × 12 mA), a hemispherical analyser operating in the fixed analyser transmission mode and the standard aperture (analysis area: 0.3 mm × 0.7 mm). The total pressure in the main vacuum chamber during analysis was typically between 10^-9^ and 10^-8^ mbar. Survey spectra were acquired at a pass energy of 160 eV. The atomic concentrations of the detected elements were calculated using integral peak intensities and the sensitivity factors supplied by the manufacturer. Binding energies were referenced to the graphitic hydrocarbon peak at 284.4 eV.

Samples were prepared by creating a bundle consisting of multiple fibres and mounting each bundle across an open cavity holder. Each sample was analysed at three different locations at a nominal photoelectron emission angle of 0° with respect to the surface normal.

FTIR

ATR spectra of the surfaces of the polymer storage media fibres were collected using a single bounce ZnSe crystal Miracle ATR accessory (Pike Technologies, Madison, USA) and a narrow band mercury cadmium telluride detector. The polymer storage materials and films cast on CaF_2_ windows were analyzed in transmission mode using a triglycine sulphate detector. All spectra were recorded at a resolution of 4 cm^-1^ with 64 scans being co-added.

SEA

Approximately 1 g of each CF sample in 300 mm lengths was packed into individual 300 mm long by 4 mm inner diameter silanized glass columns (Surface Measurement Systems, Alperton, Middlesex, UK). The estimated fibre end fraction of ~1x10^-5^ suggests that virtually all information is being obtained from the longitudinal surface of the CFs. Samples were conditioned in situ by heating for two hours at 30^o^C and 0% RH with an 8 sccm total flow rate of helium carrier gas. Use of the more severe conditions of 70 and 120^o^C were also investigated. A series of *n*-alkanes (*n*-hexane, *n*-heptane, *n*-octane and *n*-nonane) and polar molecules (chloroform, ethyl acetate, acetone, ethanol and dichloromethane) were used as probes.

The retention times were converted into retention volumes and the dispersive surface energy ($\gamma_{S}^{D}$) and specific free energy of desorption (${\Delta G}_{SP}^{0}$) on a solid sample were determined in accordance with the standard method described by Jones [^1^](#_ENREF_1). The ${\Delta G}_{SP}^{0}$ value obtained from the chloroform and ethyl acetate pair of mono-functional acidic and basic probes was used to determine the acid and base properties of the samples by applying the acid–base theory developed by van Oss [^2^](#_ENREF_2). The specific component of the surface energy ($\gamma_{S}^{AB}$) was calculated as the geometric mean of the surface energies determined for this acid (Lewis acceptor) - base (Lewis donor) pair [^3^](#_ENREF_3). Total surface energy,$\gamma_{S}^{T},$ was calculated as the sum of the dispersive ($\gamma_{S}^{D}$) and specific ($\gamma_{S}^{AB}$) energy contributions and the surface polarity was calculated as${\gamma_{S}^{AB}}/{\gamma_{S}^{T}}$. The acid and base numbers, K_a_ and K_b_, and hence the acid-base ratios (K_a_/K_b_) were determined according to the method of Gutmann [^4^](#_ENREF_4).

Fitting the data to an exponential decay function enables detailed analysis of the data [^5^](#_ENREF_5). After fitting the data with the function y = y_0_ + Ae^x/t^, the extent of heterogeneity can be assessed in terms of the range of energies (the pre-exponential factor, A) as well as the fraction of the surface coverage over which the decline occurs (proportional to the decay constant, t). From the exponential equation of fit the maximum (y_0_ + A) as well as y(1) , the average surface energy of the whole sample (fractional coverage of 1), are also obtained.

SFFT

For single fibre-composite fragmentation testing, a single CF (70 mm in length) was positioned in the centre of a mould with a dog-bone shaped cavity and a parallel region of the dimensions 25 mm × 5 mm × 1.5 mm. The two ends of the fibre extended over the mould and were attached to clips with 150 mg weights in order to hold the fibre straight in the test specimen. Epoxy resin and hardener (RIM935 and RIM937, Hexion Inc., Columbus, USA) were mixed, 100:40 by weight, and held under vacuum to remove any voids. The epoxy was then poured into the mould to completely cover the fibre and cured at room temperature for 2 days with a further post-cure at 100°C for 12 h. The specimens were then ground and polished to obtain the final test coupons.

During testing, fragmentation over the entire fibre length was monitored using a digital microscope (AD-4113ZT Dino-Lite, AnMo Electronics Co. Taiwan) and the number of fibre fragments within the gauge length of 20 mm were counted. The length of each fragment was then measured using an Olympus SZX12 long stereo zoom optical microscope fitted with polarizers and an Olympus DP70 digital camera. The apparent shear strength at the interface ($\tau_{IFSS}$) was estimated from the Kelly-Tyson model, $\tau_{IFSS}=\frac{\sigma_{f}d_{f}}{{2l}_{c}}$, where $l_{c}=\frac{3}{4}l$, $\sigma_{f}$is the fibre strength at the critical fragment length, $d_{f}$ is the fibre diameter and $l_{c}$ is the critical fragment length of the fibre which can be obtained from the mean fibre fragment length, $l,$at saturation [^6^](#_ENREF_6). The $\sigma_{f}$value is determined using the fibre tensile strength and Weibull modulus.

Tensile testing

Linear density (nominal value of 0.8 tex as per supplier specifications) was determined using a gauge length of 25 mm and a pre-tension of 1.5 cN/tex. Tensile load - extension curves were collected at a test speed of 2.0 mm/min, a gauge length of 25 mm and a pre-tension of 0.5 cN/tex. Load data was normalized by dividing by the linear density to give specific stress strain curves from which tensile strength (ultimate specific stress or tenacity) could be determined.

**Supplementry Tables**

**Table S1** XPS elemental surface composition, expressed as atomic ratio (X/C), obtained from the stored oxidized CF samples.

|  | Spool | | Bag, 7 days | | Bag, 2 months | | Spool,  DCM rinsed | | Bag,  DCM rinsed | |
| --- | --- | --- | --- | --- | --- | --- | --- | --- | --- | --- |
|  | **Mean** | **SD** | **Mean** | **SD** | **Mean** | **SD** | **Mean** | **SD** | **Mean** | **SD** |
| Na 1s | 0.000 | 0.000 | 0.000 | 0.000 | 0.000 | 0.000 | 0.002 | 0.001 | 0.003 | 0.000 |
| O 1s | 0.079 | 0.001 | 0.079 | 0.000 | 0.078 | 0.000 | 0.081 | 0.000 | 0.087 | 0.002 |
| N 1s | 0.048 | 0.001 | 0.047 | 0.003 | 0.050 | 0.001 | 0.047 | 0.002 | 0.050 | 0.001 |
| C 1s | 1.000 | 0.000 | 1.000 | 0.000 | 1.000 | 0.000 | 1.000 | 0.000 | 1.000 | 0.000 |
| Cl 2p | 0.001 | 0.000 | 0.000 | 0.000 | 0.000 | 0.000 | 0.001 | 0.000 | 0.001 | 0.000 |
| Si 2p | 0.001 | 0.000 | 0.001 | 0.000 | 0.002 | 0.000 | 0.001 | 0.000 | 0.002 | 0.000 |

**Table S2** Parameters obtained from fitting an exponential decay function; y = y_0_ + Ae^x/t^, to the surface energy data pre-treated spool and zip-lock bag stored CFs.

| Energy Type | CF Storage  and Pre-treatment Conditions | Energy (mJ/m^2^) | | | Decay Constant (t) |
| --- | --- | --- | --- | --- | --- |
|  |  | **Average y(1)** | **Maximum y(0)** | **Range (A)** |  |
| Dispersive  ($\boldsymbol{\gamma}_{\boldsymbol{S}}^{\boldsymbol{D}}$) | Spool, conditioned at 30^o^C | 40.7 | 100.2 | 59.5 | 0.093 |
|  | Spool, conditioned 120^o^C | 42.0 | 90.1 | 48.0 | 0.071 |
|  | Spool, DCM rinsed, conditioned 30^o^C | 50.8 | 112.8 | 62.0 | 0.048 |
|  |  |  |  |  |  |
|  | Bag, 1 month, conditioned at 30^o^C | 45.0 | 77.0 | 32.0 | 0.046 |
|  | Bag 1 month, conditioned 120^o^C | 40.5 | 81.3 | 40.8 | 0.050 |
|  | Bag for 1 month, DCM rinsed, conditioned 30^o^C | 53.6 | 112.5 | 59.0 | 0.044 |
| Specific  ($\boldsymbol{\gamma}_{\boldsymbol{S}}^{\boldsymbol{AB}}$) | Spool, conditioned at 30^o^C | 23.1 | 61.4 | 38.2 | 0.063 |
|  | Spool, conditioned 120^o^C | 18.3 | 52.7 | 34.4 | 0.064 |
|  | Spool, DCM rinsed, 30^o^C conditioned | 26.2 | 61.3 | 35.1 | 0.041 |
|  |  |  |  |  |  |
|  | Bag, 1 month, | 21.2 | 49.4 | 28.2 | 0.046 |
|  | Bag for 1 month, conditioned 120^o^C | 18.6 | 49.0 | 30.4 | 0.050 |
|  | Bag 1 month, DCM rinsed, 30^o^C conditioned | 25.9 | 59.7 | 33.8 | 0.044 |
| Total  ($\boldsymbol{\gamma}_{\boldsymbol{S}}^{\boldsymbol{T}}$) | Spool, conditioned at 30^o^C | 64.7 | 161.2 | 96.5 | 0.079 |
|  | Spool, 120^o^C | 60.4 | 142.8 | 82.4 | 0.068 |
|  | Spool, DCM rinsed, 30^o^C conditioned | 77.0 | 174.0 | 96.9 | 0.045 |
|  |  |  |  |  |  |
|  | Bag, 1 month, conditioned at 30^o^C | 66.1 | 126.3 | 60.2 | 0.046 |
|  | Bag 1 month, 120^o^C | 59.1 | 130.3 | 71.2 | 0.050 |
|  | Bag 1 month, DCM rinsed, 30^o^C conditioned | 79.5 | 172.2 | 92.7 | 0.044 |

**Table S3** Single fibre-composite fragmentation results for stored sized CFs

| **CF Storage** | **Tensile Strength**  **(cN dtex**^-1^**)** | | **Weibull Modulus** | **Fragment Length (µm)** | | $\tau_{\mathrm{IFSS}}$  **(cN dtex**^-1^**)** |
| --- | --- | --- | --- | --- | --- | --- |
|  | **Mean** | **SD** |  | **Mean** | **SD** |  |
| **Spool** | 21.89 | 3.27 | 7.84 | 378.47 | 91.76 | 0.268±0.075 |
| **Bag** | 21.77 | 3.40 | 7.79 | 376.10 | 96.54 | 0.270±0.076 |

**Supplementary Figures**

**Figure S1.** Infrared transmission spectra obtained from the storage media: (a) shrink-wrap used to protect spools of carbon fibre and (b) zip-lock bag used to store the carbon fibres.

Very weak features (relative intensities indicative of concentrations much less than 1%) were observed at 1742, 1715 and 1645 cm^-1^ in the spectrum obtained from the shrink wrap, while significantly more intense bands were observed at 1742 and 1645 cm^-1^ in that of the zip-lock bag. Due to their weakness, these features cannot be associated with the presence of copolymers. They more likely can be associated with additives or surface finishes.

**Figure S2.** Infrared spectra obtained from films cast from the DCM rinse residues obtained from (a) sized CF from spool and (b) sized CF stored in a zip-lock bag for 6 months.

**
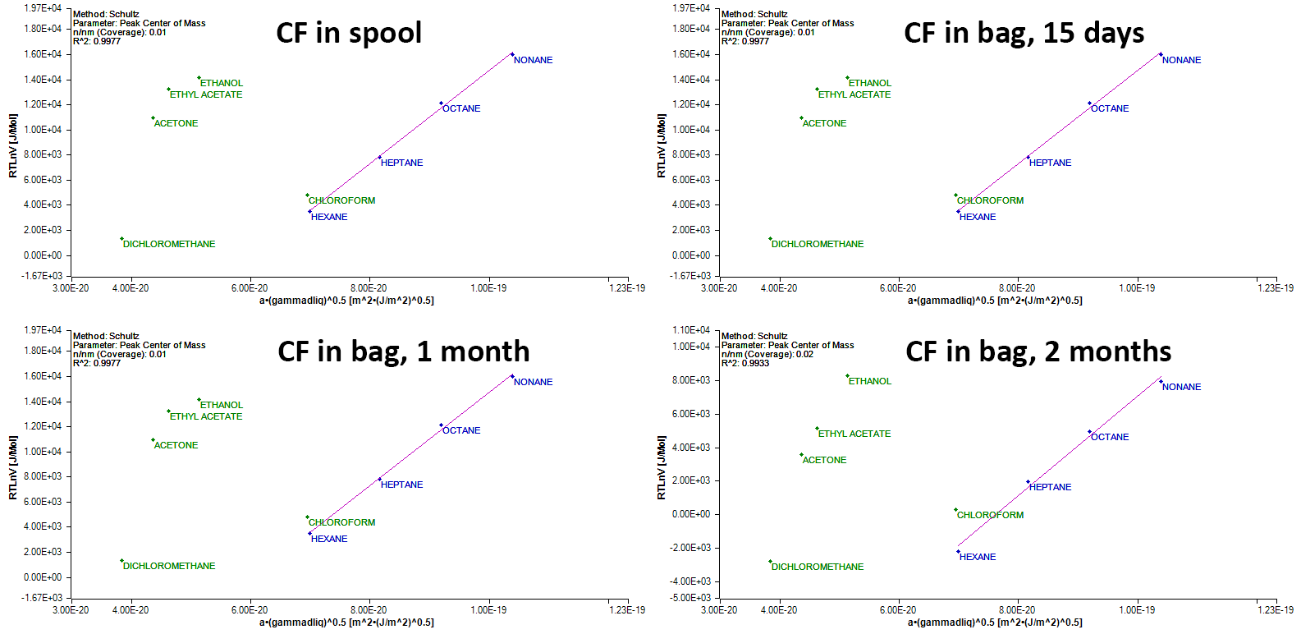
**

**Figure S3.** Graphical depiction of the Schultz method followed to obtain the specific contributions of the adsorption free energy to the total values measured for the different polar probes. The solid line corresponds to the n-alkane trend line.

**



**

**Figure S4.** Time sequences depicting the change in dispersive surface energy (a) and specific (acid–base) surface energy (b) for the oxidized CFs pre-conditioned at 30^o^C.

**Appendix**

**Appendix A.**

**Depth of penetration of ATR FTIR experiments**

It has been shown that the depth of penetration (when the depth at which the evanescent wave intensity drops off to 1/e of its initial value) is directly proportional to the wavelength of light and inversely proportional to the sine squared of the angle of incidence and the square of the ratio of the refractive index of the internal reflective element (IRE) to that of the sample [^7^](#_ENREF_7). Ohwaki and Ishida in their optimization of data collection parameters for the surface characterization of carbon fibres by infrared ATR spectroscopy found that a Ge IRE with its refractive index of 4.00 best matched that of pure carbon based materials [^8^](#_ENREF_8). The refractive index of Ge asymptotically approaches 4.00 at wavelength values above 1200 nm (833 cm^-1^) [^9^](#_ENREF_9). For highly absorbing materials such as carbon fibres, the use of the Ge IRE has the added benefit of limiting the pathlength of the evanescent wave.

The real part (n) of the complex refractive index (n - ik) of high tenacity carbon fibre was determined by Freitag and co-workers [^10^](#_ENREF_10) to be 2.3 at 796 nm. This is in good agreement with the refractive indexes of amorphous carbon (2.434) and pyrolytic graphite (2.767) as determined at 790 nm [^11^](#_ENREF_11). It was found that the refractive index of these materials decreased upon oxidation [^11^](#_ENREF_11) making a value of 2.3 very reasonable for oxidized carbon fibre. Using this value for carbon fibre and a value of 4.0 for Ge, the depth of penetration into the fibre at 1700 cm^-1^ (C=O stretching region) can be calculated to be 570 nm while at 3000 cm^-1^ (C-H stretching region) the penetration is reduced to 320 nm. This is 30 to 50 times greater than what is typical for XPS analysis (see Appendix B below).

Following on from the argument presented in Appendix B, the analysis at these depths would suggest that the signal due to the contamination layer at 1700 cm^-1^ would be diluted by a factor of the order of 100 more than in an XPS experiment (1 molecular layer of comtanination on top of 1000 basal plane layers)

**Appendix B.**

**The sensitivity of XPS experiments**

Since the actual emission angle is ill-defined in the case of samples with a rough or rounded surface (ranging from 0º to 90º) the sampling depth may range from 0 to approximately 10 nm. In the latter case, if the carbon fibre surface is represented as a stack of basal planes with a separation of 0.3354 nm, any surface active species would only be present on the outer most plane of a stack of 30 [^12^](#_ENREF_12). The sensitivity of XPS can be increased by decreasing the angle between the detector and the sample. An angle of 50^o^ will decrease the analysis depth to ~4.5 nm or 14 planes, which is still quite significant compared to the atom layer probed by SEA. Tilting the sample however has the negative effect of lowering the counts obtained from a sample and thus data collection time has to be significantly extended.

While the XPS analysis carried out on carbon fibre often utilizes detector geometries normal to the sample surface [^13^](#_ENREF_13)^,^[^14^](#_ENREF_14), work also has been carried out using lower angles [^15^](#_ENREF_15)^,^[^16^](#_ENREF_16). Only a few papers however compare the results obtained at more than one angle [^17^](#_ENREF_17)^,^[^18^](#_ENREF_18). In one particular study nitrogen was detected on the surface of carbon fibres that were coated with a size that contained no nitrogen atoms. This nitrogen was attributed to the underlying carbon fibre as it was found that as the detector angle was decreased the nitrogen signal also decreased [^17^](#_ENREF_17)^,^[^18^](#_ENREF_18). However, statistical analysis of data obtained from a number of spots suggests that this decrease is not statistically significant at the 95% confidence limits. As the surface contamination present on our samples is much thinner, as well as more unevenly dispersed, when compared to that of a typical sizing layer we would not expect angle resolved XPS to significantly improve the ability to detect the surface contamination present on our samples.

In comparing XPS results to other techniques, it should be pointed out that XPS is an ultra-high vacuum (UHV) technique and under such conditions it is possible for the surface of a material to change. Desimoni and co-workers found that storage in UHV can give rise to the chemical modification of the CF surface and in particular contaminants can be desorbed from the surface [^19^](#_ENREF_19). Dynamic surfaces can change in response to the UHV environment and it is also possible that an ultraclean surface can pick up hydrocarbons from the vacuum system and thus become more dispersive in nature.

**References**

1 Jones, M. D., Young, P. & Traini, D. The use of inverse gas chromatography for the study of lactose and pharmaceutical materials used in dry powder inhalers. *Adv Drug Deliv Rev*. **64**, 285-293, (2012).

2 Van Oss, C. J. *Interfacial forces in aqueous media*. (CRC press, 2006).

3 Traini, D., Young, P. M., Thielmann, F. & Acharya, M. The influence of lactose pseudopolymorphic form on salbutamol sulfate-lactose interactions in DPI formulations. *Drug development and industrial pharmacy*. **34**, 992-1001, (2008).

4 Gutmann, V. *The donor-acceptor approach to molecular interactions*. (Plenum Press, 1978).

5 Huson, M. G. *et al.* Heterogeneity of carbon fibre. *Carbon*. **68**, 240-249, (2014).

6 Kelly, A. & Tyson, W. R. Tensile properties of fibre-reinforced metals: Copper/tungsten and copper/molybdenum. *Journal of the Mechanics and Physics of Solids*. **13**, 329-350, (1965).

7 Harrick, N. J. *Internal Reflection Spectroscopy*. (Interscience Publishers, 1967).

8 Ohwaki, T. & Ishida, H. Optimization of the surface characterization of carbon fiber by FT-IR internal reflection spectroscopy. *Appl Spectrosc*. **49**, 341-348, (1995).

9 Icenogle, H. W., Platt, B. C. & Wolfe, W. L. Refractive indexes and temperature coefficients of germanium and silicon. *Appl Opt*. **15**, 2348-2351, (1976).

10 Freitag, C., Weber, R. & Graf, T. Polarization dependence of laser interaction with carbon fibers and CFRP. *Opt Express*. **22**, 1474-1479, (2013).

11 Stagg, B. J. & Charalampopoulos, T. T. Refractive indices of pyrolytic graphite, amorphous carbon, and flame soot in the temperature range 25° to 600°C. *Combust Flame*. **94**, 381-396, (1993).

12 Jain, M. & Abhiraman, A. Conversion of acrylonitrile-based precursor fibres to carbon fibres. *J Mater Sci*. **22**, 278-300, (1987).

13 Alexander, M. R. & Jones, F. R. Effect of electrolytic oxidation upon the surface chemistry of type A carbon fibres: III. Chemical state, source and location of surface nitrogen. *Carbon*. **34**, 1093-1102, (1996).

14 Servinis, L., Gengenbach, T. R., Huson, M. G., Henderson, L. C. & Fox, B. L. A Novel Approach to the Functionalisation of Pristine Carbon Fibre Using Azomethine 1,3-Dipolar Cycloaddition. *Aust J Chem*. **68**, 335-344, (2015).

15 Alexander, M. R. & Jones, F. R. The chemical environment of nitrogen in the surface of carbon fibres. *Surf Interface Anal*. **22**, 230-235, (1994).

16 Weitzsacker, C. L., Xie, M. & Drzal, L. T. Using XPS to Investigate Fiber/Matrix Chemical Interactions in Carbon-fiber-reinforced Composites. *Surf Interface Anal*. **25**, 53-63, (1997).

17 Dilsiz, N. & Wightman, J. P. Surface analysis of unsized and sized carbon fibers. *Carbon*. **37**, 1105-1114, (1999).

18 Reis, M. J., Botelho Do Rego, A. M., Lopes Da Silva, J. D. & Soares, M. N. An XPS study of the fibre-matrix interface using sized carbon fibres as a model. *J Mater Sci*. **30**, 118-126, (1995).

19 Desimoni, E., Casella, G. I., Salvi, A. M., Cataldi, T. R. I. & Morone, A. XPS investigation of ultra-high-vacuum storage effects on carbon fibre surfaces. *Carbon*. **30**, 527-531, (1992).
